# Plasticity-first evolution via CYP405 loss shaped chemical defenses in butterflies

**DOI:** 10.1093/molbev/msag197

**Published:** 2026-08-10

**Authors:** Erika C Pinheiro de Castro, Francesco Cicconardi, Ian A Warren, Nicol Rueda-M, Camilo Salazar, Søren Bak, Stephen H Montgomery, Chris D Jiggins

**Affiliations:** Department of Zoology, University of Cambridge, Camrbidge, UK; School of Health, Sciences and Society, University of Suffolk, Ipswich, UK; School of Biological Sciences, University of Bristol, Bristol, UK; Department of Genetics and Evolutionary Biology, Bioscience Institute, University of São Paulo, São Paulo, Brazil; Department of Zoology, University of Cambridge, Camrbidge, UK; Tree of Life Programme, Wellcome Sanger Institute, Hinxton, UK; School of Sciences and Engineering, Biology Program, Universidad del Rosario, Bogotá, Colombia; School of Sciences and Engineering, Biology Program, Universidad del Rosario, Bogotá, Colombia; Department of Plant and Environmental Science, University of Copenhagen, Frederiksberg, Denmark; Department of Zoology, University of Cambridge, Camrbidge, UK; School of Biological Sciences, University of Bristol, Bristol, UK; Department of Zoology, University of Cambridge, Camrbidge, UK

**Keywords:** phenotypic plasticity, cyanogenic glucosides, *Heliconius*, cytochrome P450, convergent evolution

## Abstract

Phenotypic plasticity allows a single genotype to maintain its fitness across different environments. This facilitates colonization of new niches but can be further refined or even lost as lineages diversify. The toxic Heliconiini butterflies have biochemical plasticity: they either sequester their cyanogenic glucosides (CGs) from their larval host plant or biosynthesize them when compounds for sequestration are not available. Here, we trace the evolution of CG biosynthesis in Heliconiini butterflies, a fundamental component of this biochemical plasticity. We first reconstructed the evolutionary history of biochemical plasticity in Heliconiini using chemical data from over 700 individuals, demonstrating that plasticity was ancestral in the tribe but subsequently lost in a few clades, such as the Sapho clade specialized in CG sequestration. In lepidopterans, CG biosynthesis has previously been characterized in the moth *Zygaena filipendulae*, as the genes *CYP405A2*, *CYP332A3*, and *UGT33A1*. Thus, we CRISPR-edited *CYP405* in *Heliconius erato* and confirmed that *CYP405*-knockout caterpillars do not biosynthesize CGs. We identified the *CYP405A*s and *CYP332A*s in other lepidopterans and found that both genes were independently co-opted into CG biosynthesis in the Heliconiinae butterflies and *Zygaena* moths. While most lepidopterans have a *CYP332A*, *CYP405A* is mostly restricted to butterflies and has been duplicated in all *Heliconius* species. Although several *CYP405A* copies were found in the Sapho clade, most of them lack structurally important P450 domains, which explains the loss of biochemical plasticity via specialization in CG sequestration. This is one of the few examples of plasticity-first evolution in which the genetic mechanisms associated with its refinement are known.

## Introduction

Phenotypic plasticity can allow species to maximize fitness across different environments, as a single genotype can produce multiple condition-dependent phenotypes. Such plasticity can also promote diversification under the “plasticity first” hypothesis, in which ancestral plasticity facilitates the colonization of novel habitats and niches, but can be further lost as lineages specialize locally (Levis and Pfennig 2019). Although most fitness traits have some degree of plasticity, phenotypic plasticity has been mainly studied in morphological and physiological traits (Sommer 2020). Chemical traits are fine-tuned by the environment and offer an excellent opportunity to exploit the molecular bases of phenotypic plasticity, promoting and maintaining diversity.

Chemical defenses create an “enemy-free” niche for protected organisms to radiate and speciate (Ehrlich and Raven 1964). Yet, chemically protected species need to evolve not only mechanisms to acquire toxins but also to tolerate/resist them, and both mechanisms need to be present for toxicity to translate into an adaptive advantage (Beran et al. 2019). To make the evolutionary trajectory of chemical defenses even more complicated, most mechanisms to acquire and tolerate toxins are biochemical pathways—such that all substrates, enzymes, and intermediates need to be present for the toxin to be formed. Chemical defense is therefore a complex trait that may be difficult to evolve. As such, gene co-option (Petschenka and Agrawal 2016) and horizontal gene transfer (Piel et al. 2004; Wybouw et al. 2016) can facilitate the acquisition of toxicity. Furthermore, the evolution of toxicity and its complexity extend beyond genetics; for most heterotrophic organisms, diet also plays a crucial role, by either providing toxins that can be directly sequestered or the precursors for their biosynthesis (Nishida 2014). This implies that: (i) the evolution of toxicity often cannot be separated from the evolution of diet specialization and (ii) there is substantial phenotypic plasticity in animal toxicity in response to diet, although this is rarely considered as an example of adaptive plasticity.

Cyanide is a toxin that disrupts the mitochondrial electron transport chain and is a breakdown product of cyanogenic glucosides (CGs). Some Lepidoptera have evolved the ability to detoxify cyanide and feed on plants protected by CG (Zagrobelny et al. 2018). While lepidopteran adaptations to detoxify cyanide came from both horizontal gene transfer (ie β-cyanoalanine synthase—Wybouw et al. 2014) and co-option of genes with existing functions (ie Rhodanase—Steiner et al. 2018), the evolution of the mechanisms used to acquire CG is less well understood. Possibly as a counter-adaptation against these specialized lepidopterans, some plant lineages/populations have disabled sequestration by changing the structure of their CGs—eg certain *Passiflora* species against heliconiine butterflies (Pinheiro de Castro et al. 2020)—or even by becoming acyanogenic—eg *Turnera ulmifolia* in Mexico against *Euptoieta* butterflies (Schappert and Shore 1999); and *Lotus corniculatus* against *Zygaena* moths (Jones 1962). These changes in the CG composition of the host plant affect the biochemical phenotype of lepidopterans that sequester these toxins. In return, some lepidopterans have evolved mechanisms to maintain their toxicity regardless of the CG profile of their host plants by biosynthesizing their own CGs.

**Figure 1:**
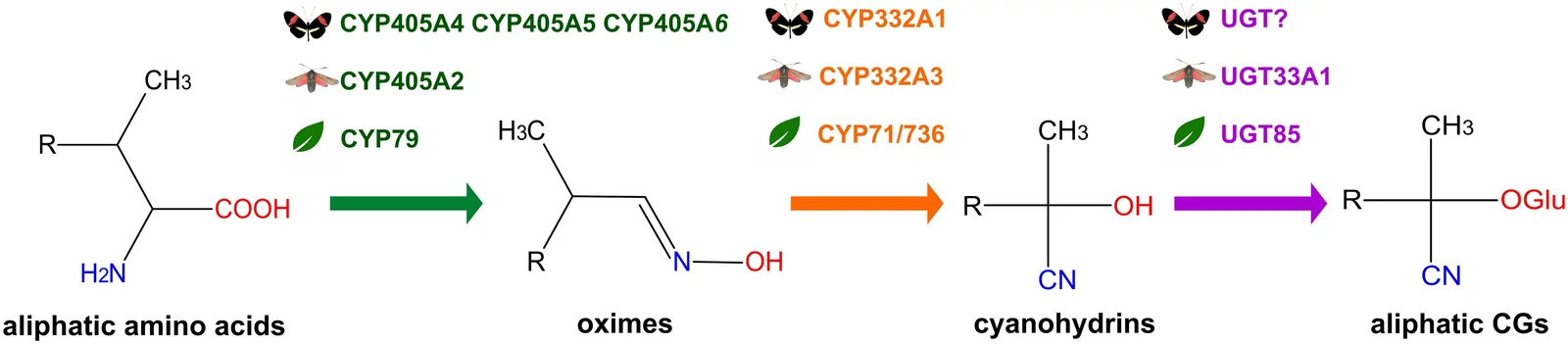
*De novo* biosynthesis of aliphatic cyanogenic glucosides (CGs) in *Heliconius melpomene* butterflies (Chauhan et al. 2013), *Zygaena filipendulae* moths (Jensen et al. 2011), and plants, such as *Lotus* and cassava. The pathway in *Heliconius* was identified by homology with *Z. filipendulae*. The colors of the arrows correspond to the three catalytic steps of CG biosynthesis and match the color of the enzyme names that catalyze them. This pathway has the same enzymatic steps in both plants and lepidopterans because of convergent evolution (Jensen et al. 2011). The radical R in the chemical structures corresponds to either CH_3_ or CH_2_-CH_3_.

Both higher plants and lepidopterans de novo biosynthesize CGs using the same three enzymatic steps: they convert amino acids into oximes and then into cyanohydrins, which are then stabilized with a glycosylation—forming a CG (Jensen et al. 2011) (Fig. 1). To assemble this pathway, cytochromes P450 (P450s/CYPs) and Uridine Diphosphate-glycosyltransferases (UGTs) have been recruited (Fig. 1). Despite having the same precursors, intermediates, products, and catalytic reactions, de novo biosynthesis of CGs evolved independently in lepidopterans and plants (Jensen et al. 2011). Moths of the family Zygaenidae and butterflies of the subfamily Heliconiinae are the only insects currently known to de novo biosynthesize CGs, and both clades produce the same aliphatic compounds. In lepidopterans, CG biosynthesis has been fully characterized in the six-spot burnet moth *Zygaena filipendulae* (Jensen et al. 2011) and is composed of CYP405A2, CYP332A3, and UGT33A1. While no homolog of *UGT33A1* was found in the genome of the butterfly *Heliconius melpomene*, this species had three *CYP405As* and one *CYP332A: HmCYP405A4*, *HmCYP405A5, HmCYP405A6,* and *HmCYP332A1* (Chauhan et al. 2013). These genes have all the conserved domains/motifs of a functional P450 and are expressed in life stages of intense CG biosynthesis (Pinheiro de Castro et al. 2020), but their involvement in CG biosynthesis in heliconiines has not yet been empirically confirmed. Since moths and butterflies split ∼100 MYA (Million Years Ago) (Kawahara et al. 2019), if CG biosynthesis in these two clades involves orthologous genes, it is likely that these were independently co-opted to this pathway, but this hypothesis remains to be tested.

While biosynthesis of CG allows Heliconiini butterflies to maintain their toxicity regardless of host plant chemistry, it may have higher fitness costs than CG sequestration. Cyanogenic lepidopterans only biosynthesize CGs when they cannot sequester these compounds during larval feeding (Fürstenberg-Hägg et al. 2014; Pinheiro de Castro et al. 2021). The CGs sequestered by heliconiines, which are cyclopentenic, have a different structure from those that are biosynthesized, which are aliphatic (Pinheiro de Castro et al. 2018). The difference in structure between sequestered and synthesized compounds enables the use of target metabolomics to identify which strategy wild-caught heliconiine individuals use to acquire their CG reservoir (Pinheiro de Castro et al. 2019). This creates a unique opportunity to study the importance of phenotypic plasticity in the evolution of toxicity. Moreover, phenotypic plasticity can itself evolve when selection acts to change reaction norms. The different degrees of host plant specialization within Heliconiini, in which different clades specialize on specific subgenera of *Passiflora*, with some feeding on several host plants, while others are completely monophagous, create a unique system in which to understand how specialization affects biochemical plasticity (Pinheiro de Castro et al. 2018). Finally, heliconiine butterflies have been studied for over 150 years, with recent efforts resulting in assembled reference genomes (Cicconardi et al. 2023) and target-metabolomics data for most species of the tribe (Pinheiro de Castro et al. 2019; Sculfort et al. 2020), which allows for powerful comparative analyses to reveal the genetic basis of the maintenance and loss of biochemical plasticity.

We first used target-metabolomic data for over 700 individuals (Pinheiro de Castro et al. 2019; Sculfort et al. 2020; Rueda-M 2023) to evaluate the importance of biosynthesis versus sequestration in each clade and reconstruct the ancestral path of CG acquisition. We then used the gene-editing technology CRISPR-Cas9 to confirm the role of *CYP405As* in CG biosynthesis in *Heliconius*. Next, we explored the evolutionary history of these genes across 58 heliconiine genomes (Cicconardi et al. 2023) and used molecular evolution analysis to test for selection on specific branches of the phylogenetic tree. We hypothesize that *CYP405As* are only duplicated in *Heliconius* that are more toxic than other heliconiines and that these copies might have been lost in some *Heliconius* clades that have specialized in sequestration and have lost biochemical plasticity.

## Results

### Biochemical plasticity is ancestral in Heliconiini butterflies but might have been lost in a few lineages

**Figure 2:**
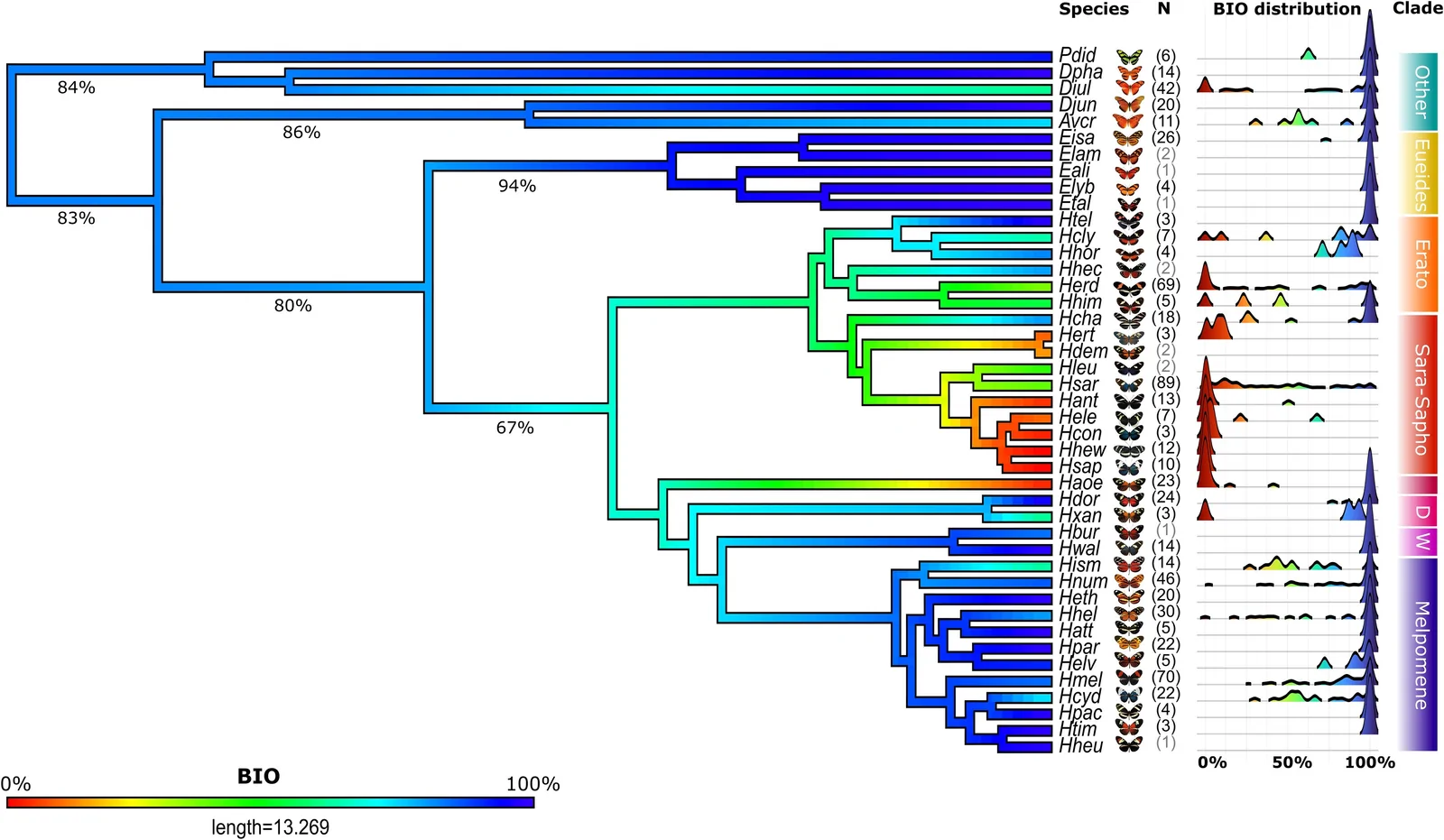
Ancestral reconstruction of biochemical plasticity in the Heliconiini tribe. This analysis used the tree hypothesis from Cicconardi et al. (2023) and a chemical dataset (743 butterflies) built based on the raw data from Pinheiro de Castro et al. 2019, Sculfort et al. (2020), and Rueda et al. (2023). The colors of the branches correspond to the estimated percentage of biosynthesized CGs (from 0% in red to 100% in blue) in the tree internal nodes based on the average per species. N corresponds to the number of samples per species, and it is followed by the density distribution of percentage of biosynthesized (blue) versus sequestered (red) CGs per species. Legend: OTHER genera—*Pdid* = *Philaethria dido; Dpha* = *Dryadula phaetusa; Diul* = *Dryas iulia; Djun* = *Dione juno; Avcr* = *Agraulis vanillae. genus EUEIDES—Eisa* = *E. isabella; Elam* = *E. lampeto; Eali* = *E. aliphera; Elyb* = *E. lybia; Etal* = *E. tales. genus Heliconius: ERATO—Htel* = *H. telesiphe; Hcly* = *H. clysonymus; Hhor* = *H. hortense; Hhec* = *H. hecalesia; Herd* = *H. erato; Hhim* = *H. himera; SARA-SAPHO: Hcha* = *H. charithonia; Hert* = *H. eratosignis; Hdem* = *H. demeter; Hleu* = *H. leucadia; Hsar* = *H. sara; H.ant* = *H. antiochus; Hele* = *H. eleuchia; Hcon* = *H. congener; Hhew* = *H. hewitsoni; Hsap* = *H. sapho. AOEDE: Haoe: H. aoede; DORIS: Hdor* = *H. doris; Hxan* = *H. xanthocles; WALLACEI: Hbur* = *H. burney; Hwal* = *H. wallacei; MELPOMENE: Hism* = *H. ismenius; Hnum* = *H. numata; Heth* = *H. ethila; Hhel* = *H. hecale; Hatt* = *H. atthis; Hpar* = *H. pardalinus; Helv* = *H. elevatus; Hmel* = *H. melpomene; Hcyd* = *H. cydno; Hpac* = *H. pachinus; Htim* = *H. timareta; Hheu* = *H. heurippa.*

We first reconstructed the evolutionary history of biochemical plasticity in the tribe Heliconiini using a phylogenetic approach, based on published CG profiles of 743 heliconiine butterflies (Fig. 2). At the base of the heliconiine phylogeny (Fig. 2, “Other genera”), CG biosynthesis is the dominant strategy. Nevertheless, sequestered CGs were found in a few individuals of all basal species (*Philaethria dido*: 1 out of 6*; Dryas iulia:* 15 out of 45; *Agraulis vanillae:* 7 out of 12; and *Dryadula phaetusa:* 1 out of 15), except for *Dione juno* (0 out of 15). This suggests that although biosynthesis is the dominant strategy, the ancestral state involved some degree of plasticity. Similarly, plasticity was also present in the ancestor of the genus *Eueides,* but biosynthesis is the most common strategy, with sequestration observed in only 2 *Eueides isabella* butterflies out of 34 *Eueides* individuals sampled, and in none of the other four species analyzed.

CG sequestration is more frequent in the ancestor of *Heliconius,* the most speciose genus of the tribe. CG sequestration was observed in almost all species within the Erato + Sara-Sapho clades, except *Heliconius telesiphe* (out of 16 species). Indeed, the ancestor of the Sara-Sapho clade favored CG sequestration over biosynthesis, and in some species, biosynthesis is rarely seen (ie *Heliconius sapho*, *Heliconius hewitsoni*, and *Heliconius antiochus*). CG sequestration was also common in the MRCA (Most Recent Common Ancestor) Aoede + Doris + Wallacei + Melpomene. *Heliconius aoede* mostly contains sequestered compounds (small amounts of biosynthesis were found in 4 out of 25 individuals). Nevertheless, CG biosynthesis regains importance in the MRCA of Doris, Wallacei and Melpomene clades, and, especially in the Melpomene clade, in which some species (4 out of 12—ie *Heliconius pachinus*, *Heliconius timareta*, *Heliconius ethila* and *Heliconius atthis*) only biosynthesize CGs and appear to have lost biochemical plasticity. Sequestered CGs were also virtually absent in *Heliconius doris* (N = 35) and *H. wallacei* (n = 14). In summary, from a plastic ancestor, some species within the genus *Heliconius* have evolved to specialize on either sequestration or biosynthesis of their CGs.

### *Heliconius erato* with CRISPR-edited *CYP405As* do not biosynthesize CGs

**Figure 3:**
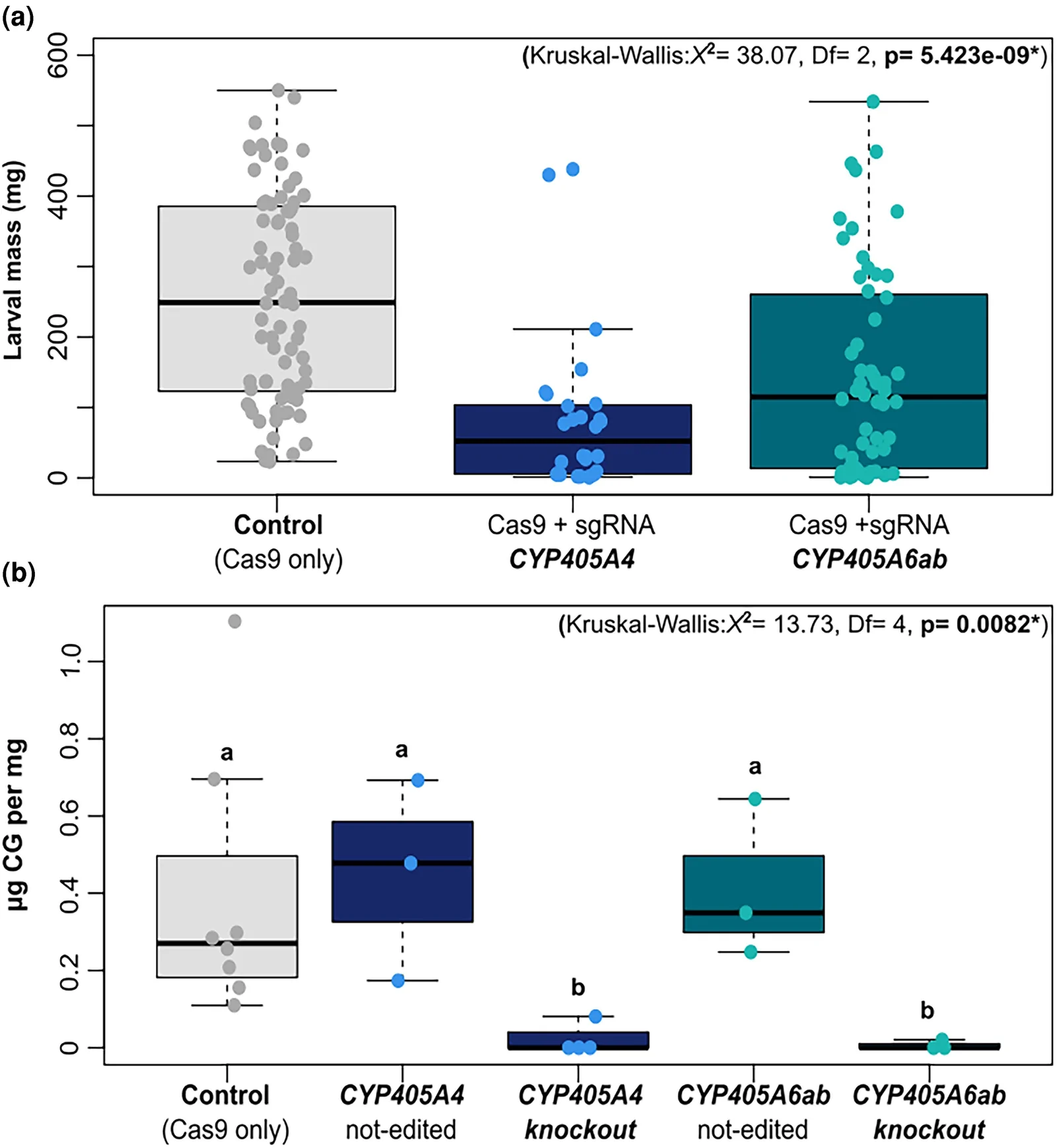
(a) Larval mass (mg) of all 7-day-old larvae from injected eggs of *H. erato*. (b) CG concentration (µg/mg) of genotyped larvae. Controls (gray) were injected with Cas9 only, whereas others were injected with Cas9 and the sgRNA specifically targeting either *CYP405A4* (navy blue) or *CYP405A6* (dark cyan). *CYP405A* knockouts had a deletion in the target copies, whereas not-edited larvae had the same genotype as control, even though they were injected with the respective sgRNAs.

We next used gene knockout experiments with CRISPR/Cas9 to confirm the role of *CYP405A* genes in CG biosynthesis. Fresh eggs of *H. erato demophoon* were injected with a single-guide RNA (sgRNA) targeting either *HedCYP405A4* or the duplicated *HedCYP405A6* (experimental group), with controls injected with just the Cas9 enzyme. Individuals in the experimental group showed reduced weight as compared to the control in the first week (Kruskal–Wallis, *X*^2^ = 38.065, degrees of freedom = 2, *P* = 5.423 × 10⁻⁹) and most failed to develop to the final instar (Fig. 3a). Target-metabolomics showed that larvae with a deletion in the CDS of *CYP405A4 or CYP405A6s* lacked or had very low biosynthesized CG in comparison with others (Fig. 3b). Larvae in the experimental group that did not have deletions in the *CYP405A4 or CYP405A6s* also had more CG than those in which gene-editing was successful. Nanopore sequencing of CRISPR-edited and control individuals also confirmed that the designed sgRNAs were specific and only caused deletions in the target *CYP405A* ortholog (Fig. S1). These results suggest that both *CYP405A4 and CYP405A6* are essential for CG biosynthesis in *H. erato.*

### *CYP405A* and *CYP332A* were co-opted twice into CG biosynthesis in Lepidoptera

To evaluate whether *CYP405As* and *CYP332As* were independently co-opted into CG biosynthesis in butterflies and moths, we first used the available *Heliconius* CYP405A4 and CYP332A1 protein sequences as a starting query to identify homologs across 63 Heliconiinae genomes, identifying 144 *CYP405A* and 66 *CYP332A* loci (Table S2). Then, we constructed a maximum likelihood tree using these Heliconiinae sequences, as well as the amino acid sequences of all complete cytochromes P450 from the CYP3 and CYP4 clans in lepidopterans (Figs. S2 and S3). Whereas *CYP332A* is present in most lepidopterans (Fig. S3), *CYP405A* is found only in a few moth species (Fig. S2). All *CYP405s* and *CYP332s* in the subfamily Heliconiinae are very conserved and are monophyletic relative to other Lepidoptera. The two *CYP405s* from *Z*. *filipendulae* and *CYP332A* cluster with butterflies rather than with other moths. Although this relationship has low bootstrap support and should therefore be interpreted cautiously, it is consistent with the hypothesis that convergent evolution in these enzymes has generated sequence similarities sufficient to affect their placement in the gene tree. We therefore tested this convergence scenario explicitly.

CSUBST analyses were performed to explicitly test for convergence in the protein-coding sequences between *Zygaena* moths and Heliconiinae butterflies. *For CYP405A*, convergent branch pairs were more frequent in *Zygaena–*Heliconiinae comparisons than in the remaining between-group comparisons (Fisher's exact test: odds ratio = 2.63, 95% confidence interval (CI) = 0.97 to 6.89, *P* = 0.0337). When evolutionary distance was taken into account in a binomial GLM, this enrichment remained positive and significant (estimate = 1.082, standard error (SE) = 0.450, *P* = 0.0161), indicating an approximately three-fold increase in the likelihood of convergence in the *CYP405As* in *Zygaena–*Heliconiinae in comparison with other Lepidoptera. The same pattern was observed for *CYP332A,* in which convergent branch pairs were more frequent in *Zygaena–*Heliconiinae comparisons than in the remaining between-group comparisons (Fisher's exact test: odds ratio = 1.78, 95% CI = 1.12 to 2.75, *P* = 0.011). This result was positive and significant even when controlling for evolutionary distance (estimate = 0.689, SE = 0.217, *P* = 0.00149), corresponding to an approximately 2-fold increase in the likelihood of convergence in the CYP332 in *Zygaena–*Heliconiinae in comparison with other Lepidoptera. We next tested whether counts of convergent substitutions were elevated in *Zygaena–*Heliconiinae branch pairs after accounting for branch length. Poisson models were overdispersed in all cases (*CYP405A: O*^N^*_C_* = 2.03, O^S^_C_ = 1.88; *CYP332A: O*^N^*_C_* = 3.92, O^S^_C_ = 4.57), so inference was based on negative binomial models with an offset of log(branch length distance). For *CYP405*, the rate of nonsynonymous convergent substitutions was significantly enriched in *Zygaena–*Heliconiinae comparisons (rate ratio, RR = 2.72, 95% CI = 1.27 to 5.83, *P* = 0.0101), as well as the rate of synonymous convergent substitutions (RR = 2.63, 95% CI = 1.62 to 4.28, *P* = 9.76 × 10^−5^). For *CYP332A*, the rate of nonsynonymous convergent substitutions (*O^N^_C_*  _any2spe_) was significantly higher in *Zygaena–*Heliconiinae than in other between-group comparisons (RR = 3.01, 95% CI = 2.06 to 4.38, *P* = 1.01 × 10^−8^). Synonymous convergent substitutions (*O^S^_C_*  _any2spe_) were also significantly elevated, although with a smaller effect size (RR = 1.88, 95% CI = 1.46 to 2.40, *P* = 6.17 × 10^−7^).

Taken together, these results indicate that branch pairs connecting *Zygaena* and Heliconiinae for both *CYP405A* and *CYP332A* exhibit a consistent enrichment of convergent substitutions relative to other lineage combinations, supporting the hypothesis of independent co-option events. This enrichment persists after accounting for evolutionary opportunity and is particularly pronounced for nonsynonymous substitutions in *CYP332A*, suggesting that adaptive convergence may contribute to the observed pattern.

### *CYP405As* duplicated in *Heliconius* and *Eueides*, but not in other heliconiines

**Figure 4:**
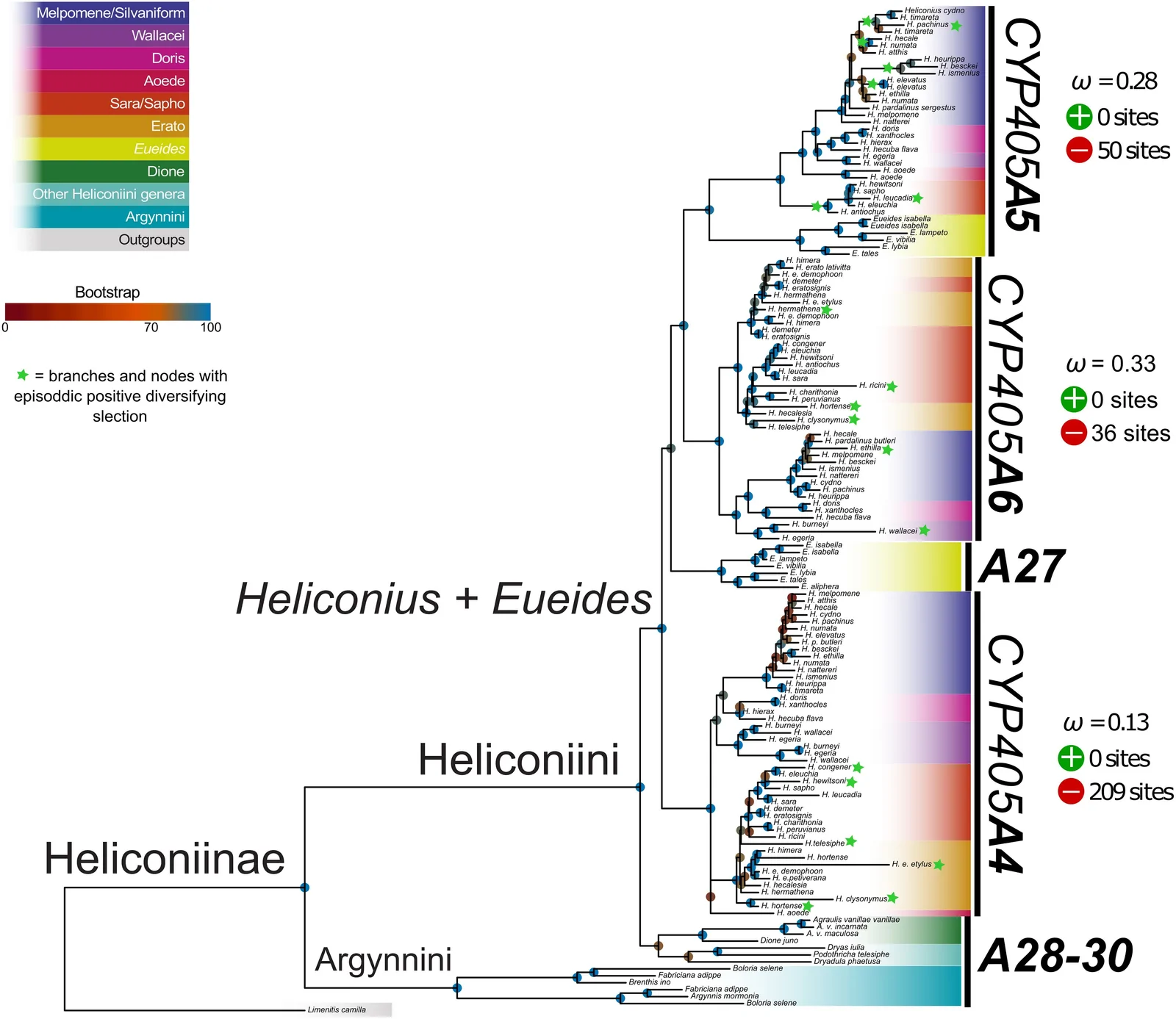
Gene-tree of *CYP405A* in butterflies of the tribes Heliconiini and Argynnini (Nymphalidae: Heliconiinae) using *Limenitis camilla* (Nymphalidae: Limenitidinae) as an outgroup. Although all Heliconiinae butterflies are predicted to be cyanogenic, Heliconiini is the only tribe for which there are substantial chemical data available. Molecular evolution analyses were performed in the Heliconiini tribe, in which purifying selection (ω) on each *CYP405* ortholog was quantified, as well as the number of sites under purifying (negative) and diversifying (positive) selection was identified. Green stars correspond to branches and nodes that experienced episodic positive diversifying selection. Colored circles correspond to branch bootstrap values (Red = low, Blue = high).

*CYP405A* was duplicated in the genera *Heliconius* and *Eueides*, but not in the other Heliconiini species (Fig. 4). A few independent duplications and losses have occurred in specific *Heliconiu*s clades/taxa, but overall, most *Heliconius* species have three or more *CYP405A*s. We hypothesize that an ancestral *CYP405A* locus was present in the outgroups (*CYP405A30*), other Heliconiini (*CYP405A29*), *Dione/Agraulis* (*CYP405A28*) and *Heliconius* (*CYP405A4*) (Fig. 4). The ancestral locus seems to have undergone a duplication (*CYP405A5*) at the MRCA of *Heliconius* and *Eueides*, followed by a loss in all the species of the *Erato* clade and in half of the ten *Sara-Sapho* clade species. The ancestral locus likely duplicated a second time at the stem of all *Heliconius* (*CYP405A6*).

The chromosomal location of *CYP405A* is highly conserved, with all copies located on ancestral chromosome 15 (chromosome 17 in *Heliconius*). Combined with the gene-tree topology, this conserved genomic location supports an evolutionary scenario involving an ancestral CYP405A locus, followed by successive duplication events at the *Heliconius* + *Eueides* ancestor and later in the *Heliconius* stem lineage.

**Figure 5:**
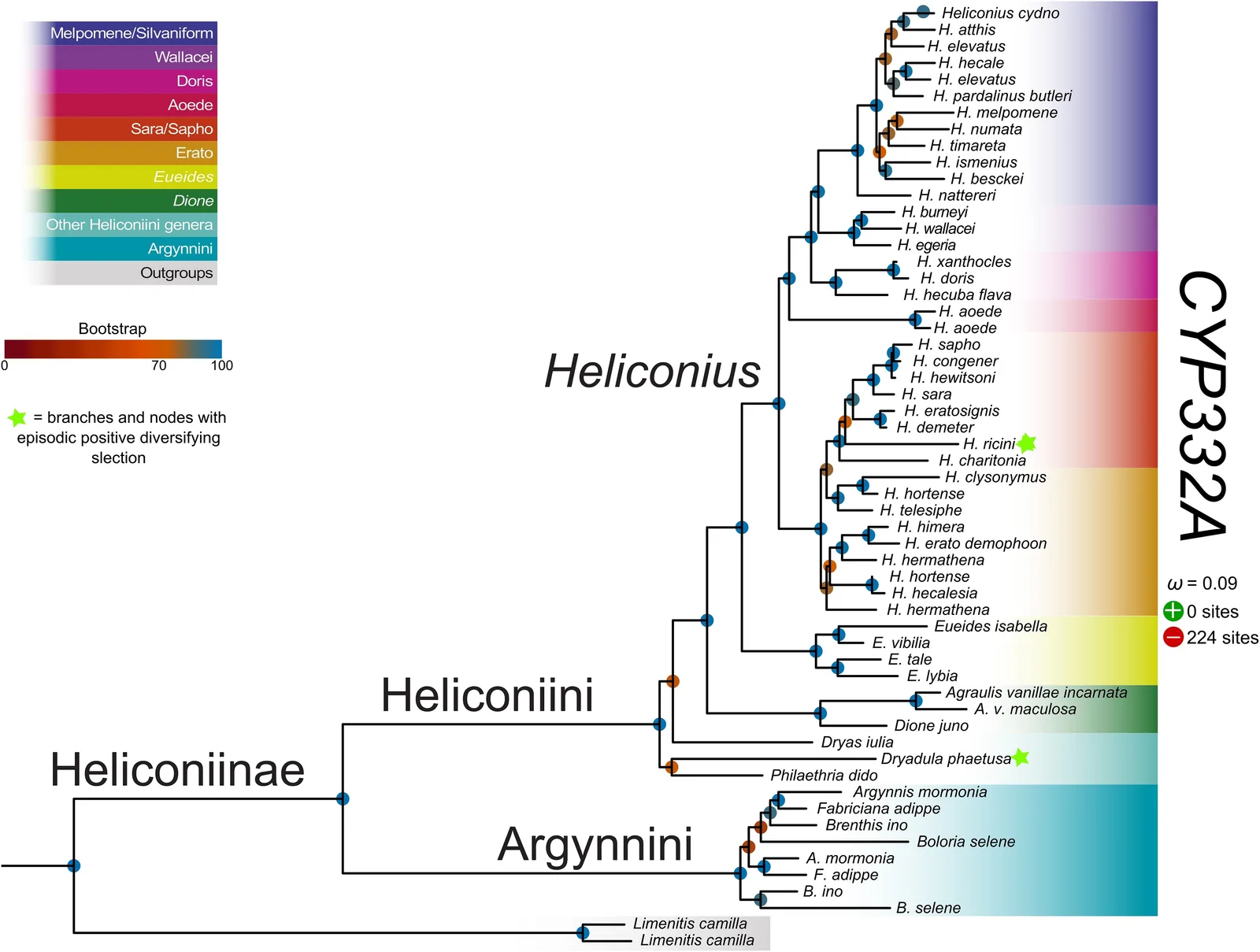
Gene-tree of *CYP332A* in butterflies of the tribes Heliconiini and Argynnini (Nymphalidae: Heliconiinae) using *Limenitis camilla* (Nymphalidae: Limenitidinae) as an outgroup. Although all Heliconiinae butterflies are predicted to be cyanogenic, Heliconiini is the only tribe for which there are substantial chemical data available. Colored circles correspond to branch bootstrap values (Red = low, Blue = high). Molecular evolution analyses were performed in the tribe Heliconiini, in which purifying selection (ω) and the number of sites under purifying (negative) and diversifying (positive) selection were identified.

*CYP332A* is the second enzyme in the CG biosynthetic pathway, and two copies of this gene were found in *Argynnis mormonia* (formerly known as *Speyeria mormonia*) and *Brenthis* (Fig. 5). The majority of Heliconiini have a single *CYP332A* locus, with the exception of *H. hortense* and *H. hermethena* (within the Erato clade), *H. aoede*, and *H. elevatus*, which have two. The locus is located on the ancestral chromosome 3 (which corresponds to chromosome 11 in *Heliconius*), without any evidence of translocations or inversions.

### Association between *CYP405A*-copy number, chemical phenotype, and host plant specialization

**Figure 6:**
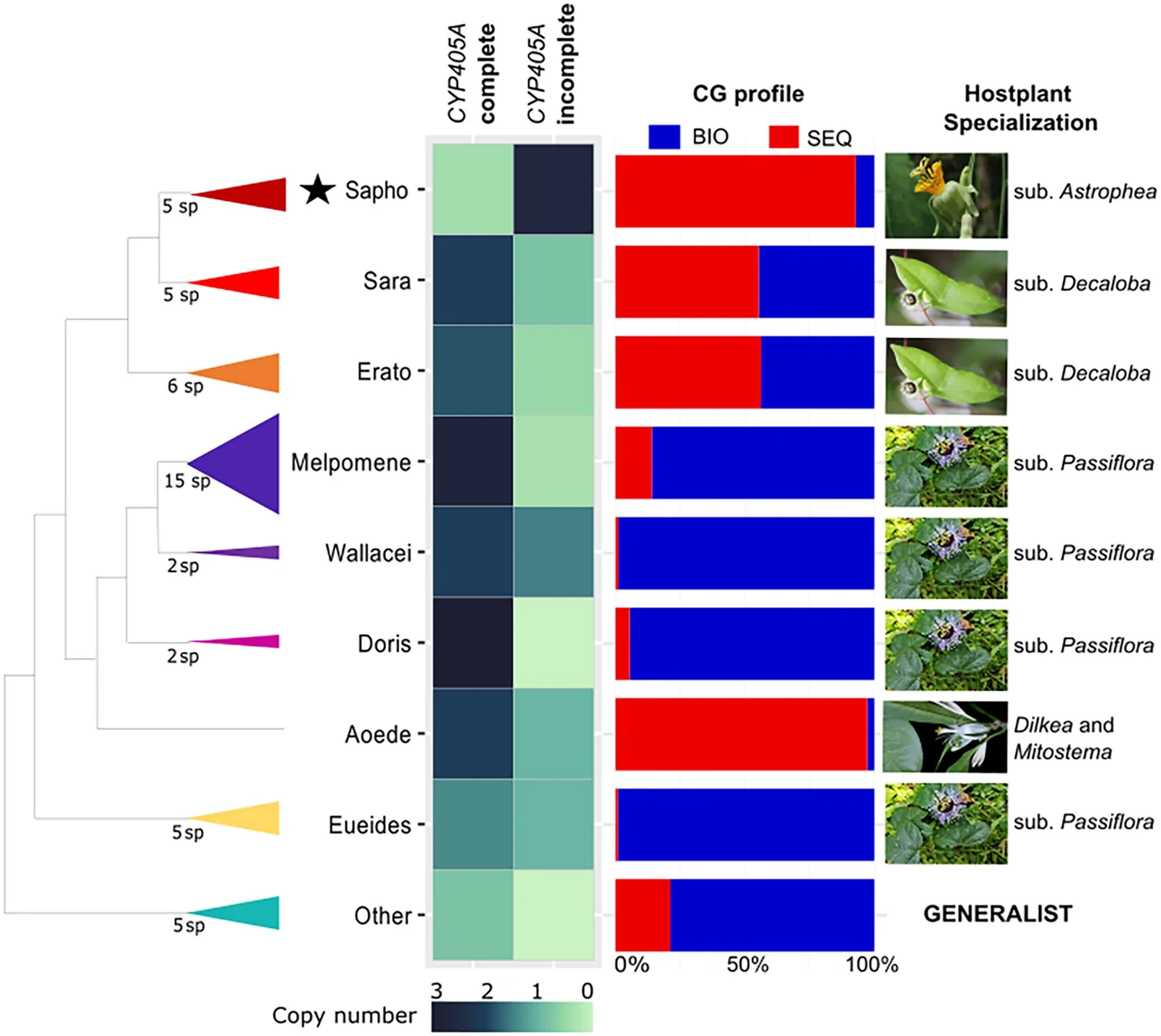
*CYP405A*-copy number, chemical profile, and host plant specialization of different Heliconiini clades. In this figure, the clade Sara-Sapho was split into two distinct clades: Sara (*H. sara*, *H. leucadia*, *H. demeter*, *H. eratosignis,* and *H. charithonia*) and Sapho (*H. sapho*, *H. antiochus, H. eleuchia, H. congener,* and *H. hewitsoni*), as they have different host plant specialization, different chemistry, and different numbers of complete *CYP405s*.

*Heliconius* species have up to four *CYP405A* genes, but some sequences were smaller than expected (∼1,500 bp). Translated sequences were therefore manually checked for the presence of the six conserved motifs in insect P450s (Feyereisen 1999): Cluster of Pro/Gly, C-Helix, I-Helix, ExLR, PERF, and Heme-binding loop. This resulted in 98 complete *CYP405As* out of 144, with incomplete copies found more frequently in the *Sapho clade* (14 incomplete sequences) (Fig. 6).

There is a clade-specific pattern in *CYP405A*-copy number, with the majority of the Silvaniform/Melpomene clade having three complete *CYP405As*, most Erato and Sara having two, and Sapho none (Fig. 6). This pattern matches the chemical profile of these clades. CG sequestration in the Heliconiini tribe gained more importance in the MRCA *Heliconius* Erato + Sara + Sapho, especially for Sapho species, and in the Aoede clade. This clade-specific pattern is also associated with their host plant specialization: while other *Heliconius* (MRCA Silvaniform/Melpomene + Doris + Wallacei) have host plants in the subgenus *Passiflora,* in which most species lack CGs that can be sequestered (Pinheiro de Castro et al. 2019), the Erato and Sara clades have host plants in the subgenus *Decaloba*, and Sapho has host plants in the subgenus *Astrophea,* where CG for sequestration is common. Aoede species use the genera *Mitostemma* and *Dilkea* as host plants from which they sequester CGs, instead of *Passiflora*. Our results therefore suggest that the loss of plasticity has also been associated with a loss of complete copies of the genes required for biosynthesis.

### P450s involved in CG biosynthesis are under purifying selection

Phenotypic plasticity might be maintained by purifying selection; therefore, we quantified the levels of purifying selection acting across the *CYP405A* orthologs and *CYP332A.* We found that *ω* varies substantially among orthologs (Figs. 4 and 5). The ancestral *CYP405* locus, *CYP405A28-30* in the outgroups and other Heliconiini and *CYP405A4* in *Heliconius*, show purifying selection (*ω* = 0.13 and *ω* = 0.17). None of the orthologs had sites under positive selection. *CYP332* shows a stronger signature of purifying selection (*ω* = 0.09; 224 sites under purifying selection; no sites under positive selection) than the *CYP405s*. We next used an adaptive branch site random effects likelihood test to identify branches that have experienced episodic diversifying positive selection. For *CYP405A*, episodic diversifying positive selection was detected in 20 out of 283 tested branches, all within the genus *Heliconius.* Most of the branches were in the Erato and Sara-Sapho clades, in which episodic diversifying positive selection was found in all ortholog groups. For *CYP332A,* episodic diversifying positive selection was only found in two branches, in *Dryadula phaetusa* and *Heliconius ricini*, in the Sara-Sapho clade.

Finally, we tested whether “sequestration-specialist” species (part of the Sara-Sapho clade) show evidence of differential selection on genes involved in CG biosynthesis. BUSTED-PH was used to test for differences in episodic diversifying positive selection between the two groups and RELAX to assess whether selection was intensified or relaxed in the target group (“sequestration-specialist”) relative to the background (“plastic species”). For *CYP405A,* we performed BUSTED-PH separately for each ortholog and detected episodic diversifying positive selection in all except *CYP405A6* in the “sequestration-specialist” group. For *CYP405A6*, episodic selection was not found in the “sequestration-specialist” group, and the RELAX test detected neither intensification nor relaxation. For *CYP332A*, episodic diversifying positive selection was also detected in both groups (“sequestration-specialist”: ω^+^ = 13.8; proportion: 1.3%; “plastic species”: ω^+^ = 10.6; proportion: 0.3%; *P* < 0.0001), with the “sequestration-specialist” showing significant intensification of selection (*K* = 1.75; *P* = 0.008).

## Discussion

Phenotypic plasticity plays an important role in allowing organisms to adapt to novel or extreme environments. However, plasticity is mainly studied in visible phenotypes. Our findings emphasize the importance of phenotypic plasticity for the evolution of toxicity, a complex molecular trait. Here, we first demonstrated, using a phylogenetic reconstruction, that there is a pattern of ancestral biochemical plasticity and derived loss of plasticity in Heliconiini butterflies, providing a novel example of plasticity-first evolution. We also use CRISPR to characterize *CYP405As* in *Heliconius erato*, demonstrating that these genes are involved in CG biosynthesis, which enables biochemical plasticity. Finally, we have shown that gene loss is associated with the loss of plasticity in a CG sequestration-specialist clade (Sapho), demonstrating a genetic mechanism for plasticity-first evolution.

Heliconiinae butterflies and *Zygaena* moths are the only lepidopterans known to biosynthesize CGs (Zagrobelny et al. 2018). Yet, most butterflies and some moths have an extra copy of *CYP405A*, suggesting that this gene was independently co-opted as the first step of CG *de novo* biosynthesis at least twice and likely has another function in other Lepidoptera (Zagrobelny et al. 2018). The high mortality and poor development of the *CYP405A*-knockout larvae in our experiments might be associated with this other function and/or the importance of CG biosynthesis for nitrogen metabolism. Similar to lepidopterans, several distantly related cyanogenic plants have co-opted *CYP79s* into the first step of CG biosynthesis (Sánchez-Pérez and Neilson 2024), while ferns have recruited a FMO (Thodberg et al. 2020). Indeed, although plants and lepidopterans biosynthesize CGs using the same enzymatic steps, the genes involved are not homologous and have evolved independently. In higher plants, different P450s have been recruited into the second step of CG biosynthesis (Takos et al. 2011; Lai et al. 2020). In lepidopterans, this second step is catalyzed by CYP332A, a gene characterized in *Zygaena* moths and found across all Heliconiini butterflies. *CYP332A* is also duplicated in the Argynnini tribe, in which cyanogenesis has been found in at least one species (Schappert and Shore 1999; Pinheiro de Castro et al. 2019). While the ancestral function of *CYP405A* in lepidopterans remains unknown, ancestral *CYP332As* were likely involved in detoxification of plant toxins (Zagrobelny et al. 2018). Most lepidopterans have one *CYP332A*, but interestingly, *Helicoverpa armigera* has two copies: while *CYP332A1* has undergone neofunctionalization and is associated with resistance to fenvalerate, the function of *CYP332A2* is still unknown (Huang et al. 2022). Biochemical constraints likely underlie the extensive co-option and repeated evolution in CG biosynthesis, highlighting the critical role of P450 gene turnover in driving diversification (Sánchez-Pérez and Neilson 2024).

Previous studies have found biosynthesis and sequestration of CGs in other Heliconiinae, which are not in the tribe Heliconiini: *Euptoieta hegesia* (tribe Argynnini—Schappert and Shore 1999; Pinheiro de Castro et al. 2019), *Cethosia cyane* (genus *Cethosia*—Pinheiro de Castro et al. 2019), and *Acraea horta* (tribe Acraeini—Raubenheimer 1989). While we hypothesize that biochemical plasticity arose in the ancestor of the whole subfamily Heliconiinae, genomic and target-metabolomics data for more species across the Heliconiinae phylogeny are necessary to test this hypothesis. Here, by reconstructing the ancestral state of biochemical plasticity in the tribe Heliconiini, we demonstrated that plasticity was ancestral in the tribe. Yet, some specific clades have undergone specialization either in CG biosynthesis (genus *Eueides* and the clade Doris) or sequestration (Erato clade), and even virtually lost biochemical plasticity (Aoede and Sapho clades). This derived accommodation of plasticity suggests that the dual strategy system used by Heliconiini to acquire their toxins represents a novel example of plasticity-first evolution—in which ancestral plasticity allows colonization of new environments/ecological niches, but the alternative phenotype can be tuned (genetic accommodation) or even become fixed (genetic assimilation/canalization) as lineages specialize in the novel conditions. Although the ability to biosynthesize CGs allowed Heliconiini butterflies to maintain their toxicity regardless of the CG composition of their larval host plants, widening their diet breadth, as lineages evolved preference for a specific host plant selection favored specialization toward one biochemical strategy over the other. The loss of plasticity in the Sapho clade is associated with the disruption of their *CYP405A*, which lacks regions that code for some of the conserved motifs that are crucial for the catalytic activity of a cytochrome P450. Species in this clade tended to have several incomplete copies of this gene and only sequester CGs. We also identified episodic diversifying selection on *CYP405A* and *CYP332A* in the Sapho clade. When a plastic phenotype is exposed to selection more often, there is greater opportunity for adaptive refinement (Lafuente and Beldade 2019). Supporting this, Sapho clade species tend to be monophagous and have a preference for a small group of *Passiflor*a in which CG for sequestration is common (Engler-Chaouat and Gilbert 2007), which could have favored the loss of biochemical plasticity in this lineage.

To date, examples of plasticity-first evolution in which the associated genetic changes are understood are scarce. For example, snowshoe hares (*Lepus americanus*) in North America often have white coloration during winter and molt to brown in spring (Jones et al. 2018). This plasticity is ancestral in the species, allowing the hares to have optimal camouflage in different seasons. Nevertheless, some populations in warmer areas, such as British Columbia, lost this plasticity and always display a winter-brown phenotype. This loss of plasticity via genetic assimilation/canalization was caused by the introgression of an *Agouti* allele from black-tailed jackrabbits to this population of snowshoe hare (Jones et al. 2018). Similarly, side-blotched lizards (*Uta stansburiana*) living on the Pisgah Volcano are cryptic and have a plastic coloration (Corl et al. 2018). The Pisgah phenotype is melanic and inhabits the lava flow, whereas the off-lava phenotype is lighter and cryptic in nearby light-colored soils. Although both lizard populations can produce the on-lava and off-lava phenotypes, genetic accommodation has led them to specialize on the phenotype that is most cryptic in their local environment. It has been shown that the Pisgah population can develop darker coloration than the off-lava populations on dark substrate, because they have specific variants of the genes *PREP* and *PRKAR1A* which control melanin formation (Corl et al. 2018). These are two clear examples of the importance of ancestral plasticity in morphological traits for the colonization of new environments, as well as the role of refinement in specific lineages toward local specialization for diversification. Our results on biochemical plasticity in Heliconiini butterflies provide another example of plasticity-first evolution in which the genetic basis for the trait is understood.

Phenotypic plasticity requires a molecular switch mechanism for sensing the environment and controlling the expression of alternative phenotypes (Sommer 2020). While biochemical plasticity is controlled by the presence of CG for sequestration in the larval host plant, how butterflies perceive these compounds in their diet and modulate the level of CG biosynthesis in response to that remains unknown. The vast majority of *Heliconius* clades evolved to have at least two complete *CYP405As.* We initially hypothesized that these were redundant orthologs, but our CRISPR experiments suggest that both *CYP405A4* and *CYP405A6* are essential for CG biosynthesis in *H. erato*. We predict that the multiple *CYP405A* copies might be necessary to fine-tune this pathway according to diet and modulate biochemical plasticity. In fact, the metabolism of toxins is often composed of enzymes that, though very similar, are not redundant and are involved in modulating responses to the environment. For example, CYP83A1 and CYP83B1 in *Arabidopsis thaliana* are very similar and catalyze the same step in the biosynthesis of glucosinolates (Bak et al. 2001; Naur et al. 2003). Despite their similarity, CYP83A1 is more efficient in using aliphatic oximes, whereas CYP83B1 is optimized for aromatic oximes, and the two orthologs enable *A. thaliana* to precisely modulate their glucosinolate profile in response to stress and developmental changes (Bak et al. 2001; Naur et al. 2003). To detoxify glucosinolates from their Brassicaceae host plants, *Pieris* caterpillars utilize the enzymes NSP (nitrile-specifier protein) and MA (major allergen) that convert these compounds into inert nitriles (Okamura et al. 2022). Although NSP and MA are derived from the same ancestor gene, NSP expression is upregulated by high concentrations of aliphatic glucosinolates, in contrast with MA, which is upregulated by aromatic glucosinolates (Okamura et al. 2019). It will be interesting to determine whether *Heliconius CYP405As* show similar complementary functions.

In summary, we have provided the first functional evidence for the genetic basis of CG biosynthesis in heliconiine butterflies and performed ancestral reconstruction analyses on their biochemical plasticity. Our results provide empirical evidence for plasticity-first evolution, with plasticity ancestral in the tribe and virtually lost in one clade specialized on sequestration. This loss was associated with genes involved in CG biosynthesis losing key functional domains.

## Methods

### CRISPR editing of *CYP405As* in *H. erato demophoon*

CRISPR-Cas9 gene editing was used to confirm that *CYP405A*, previously characterized in Z*ygaena* moths, is also involved in the de novo biosynthesis of CG in heliconiines. This experiment was performed using a population of *H. erato demophoon* kept under insectary conditions (Supplementary Methods). This species has three copies of *CYP405As*: *HedCYP405A4*, *HedCYP405A6a,* and *HedCYP405A6b*. sgRNAs targeting these genes were designed using the software Geneious (v2020.1) by identifying N_20_NGG sites within exons of the target genes and internally blasting these sequences against the genome of *H. erato demophoon* v1 (http://ensembl.lepbase.org/Heliconius_erato_demophoon_v1/Info/Index) to remove sgRNA candidates with possible off-targeting sites. sgRNA4 (GCCTAACGGTGATCCTCAGAAGG) was selected targeting a region in Exon 7 of *HedCYP40A5A4,* and sgRNA6ab (GCCTAACGGTGACCCCATGAAGG) targeting the same region in both *HedCYP405A6a* and *HedCYP405A6b.* Unfortunately, it was not possible to design an sgRNA that could distinguish between the CDS of these two orthologs due to their similarity. sgRNAs were ordered from Synthego (https://www.synthego.com/) and dissolved following the manufacturer's instructions. Freshly laid eggs of *H. erato* (within 1 to 2 h of oviposition) were glued on microscope slides and injected with a mix containing 200 ng Cas9 (TrueCut Cas9 ProteinV2, Invitrogen) with 200 ng sgRNA per µL. As a control for each batch, some eggs were glued on microscope slides and injected with Cas9 only (200 ng/µL) or kept noninjected. Larvae from control and CRISPR eggs were individually raised in tubes and fed ad libitum with *Passiflora biflora* leaves, which do not contain CGs that can be sequestered by heliconiines (forcing them to biosynthesize their own CGs) (de Castro et al. 2021). Due to high mortality in the experimental group (injected with sgRNAs), larvae were sampled after 7 to 8 d of hatching. For the phenotypic analyses, larvae were weighed on the day of sampling and part of their tissue was used for quantification of CGs using liquid chromatography-mass spectrometry (LC-MS) following the extraction and analytical methods described in Pinheiro de Castro et al. (2019). Kruskal–Wallis and Dunn’s tests were performed in R (R Core Team 2025) to test if the weight and CG content of CRISPR-edited larvae were lower than the control.

### Genotyping the CRISPR-edited *CYP405As* and control larvae

Nanopore Minion sequencing of PCR products was used for genotyping using the four-primer PCR kit (Oxford Nanopore Technologies plc., Oxford, UK; Kit: SQK-PBK004). DNA was extracted using an in-house bead extraction protocol (Kučka and Chan 2022). The following primers were used to amplify DNA from all three copies of the gene and add adapters for sequencing (Forward: TTTCTGTTGGTGCTGATATTGCAAGATTGACTCCATTGCACACT, Reverse: ACTTGCCTGTCGCTCTATCTTCCAATGACATCAGGTACATTGCG). PCR products were visualized on an agarose gel prior to sequencing library preparation and barcoding, which was performed using the manufacturer's protocol (kit: SQK-PBK004). Each sequencing run was done using partial flow cells and was stopped once all libraries in the run had 10,000 sequencing reads. Basecalling was done by the guppy basecaller (Wick et al. 2019), adapter trimming and quality control was performed using Porechopper (https://github.com/rrwick/Porechop) and quality control was performed using MinionQC.r (Lanfear et al. 2019). Minimap2 (Li, 2018) was used to align the reads to a contig containing all three gene copies. Samtools (Danecek et al. 2021) was used to convert the resulting output to a BAM file, and then to sort and index the files. The alignments were visualized in Geneious v2020.1.

### Biosynthesis versus sequestration profile of heliconiines and ancestral reconstruction analyses

Published datasets from Pinheiro de Castro et al. (2019), Sculfort et al. (2020), and Rueda-M (2023) on the CG profile of different heliconiine species were used for this analysis, comprising 743 butterflies of 43 species. The concentration of each CG (µg/mg), as well as the total CG concentration (µg/mg), found in each examined individual was extracted from the original datasets. The aliphatic CGs linamarin, lotaustralin, and epilostaustralin were labeled as “biosynthesized CG” (Table S1). The cyclopentenyl CGs deidaclin/tetraphyllin A, tetraphyllin B, epivolkenin, gynocardin, and dyhidrogynocardin were labeled as “sequestered CGs” (Table S1). The sum of the concentration of all biosynthesized CGs and all sequestered CGs in each individual was divided by its total CG concentration to calculate “% of biosynthesis and % of sequestration,” respectively. The distribution of % of biosynthesis in each species in our dataset was plotted as density plots following the tree hypothesis from (Cicconardi et al. 2023). An average of % of biosynthesis for each species in our dataset was calculated using all related individual entries and used to perform ancestral reconstruction analysis on biochemical plasticity using the function “fastANC” in the package “Phytools” (Revell 2024). This function uses Brownian motion as an underlying model to estimate ancestral node values for a continuous trait based on a phylogeny and tip states.

### Cytochrome P450 CYP405As and CYP332A: phylogeny, convergent evolution and evolutionary pressure analyses

To evaluate the phylogenetic relationship of *CYP405As* and *CYP332As* and test whether the co-opted genes in the moth *Z. filipendulae* and the butterfly subfamily Heliconiinae belonged to the same orthogroups (OGs), we first downloaded all P450 genes of the CYP4 and CYP3 clades from insectp450.net database (Wu et al. 2025) and selected all sequences from Lepidoptera annotated as complete, for a total of 9,230 sequences from 26 families. Amino-acid sequences were aligned with ClustalW and used to generate the full maximum likelihood phylogenetic tree using IQ-Tree2 (Minh et al. 2020) using the LG + G4 + F and 1,000 ultrafast bootstrap replicates. Amino acid sequences of *CYP405As* and *CYP332As* from *Heliconius melpomene* and *Z. filipendulae* were obtained from NCBI and used to identify orthologous genes within the focal clade. Once the OGs were identified, we added all those genes to our list to generate the full OG phylogenetic trees using nucleotide sequences (see below). Moreover, the protein sequences of CYP405As and CYP332As from *H. melpomene*, *H. erato*, *Bombyx mori,* and *Z. filipendulae* were used as queries in EXONERATE (Slater and Birney 2005) [setting: –n 3 –model protein2genome –percent 50 –refine full] to annotate coding exons and introns in 58 heliconiini and 2 other heliconiinae (*Argynnis mormonia* and *Brenthis ino*) species. The resultant nucleotide sequences were subsequently mapped using Minimap2 (Li, 2018) to explore genome assemblies where no record was found in the Exonerate mapping to minimize false negative hits. All the amino acid sequences obtained were processed with the CDSearch webserver (Lu et al. 2020) and the BLASTp webserver to verify the presence and the completeness of the conserved domain (Pfam00067) and possible gene fusions. All nucleotide sequences were aligned using MACSE2 (Ranwez et al. 2018), and an initial maximum likelihood phylogenetic gene tree reconstruction was generated as implemented in IQ-Tree2 [-m MFP -B 5000]. At this point, all sequences with putative stop codons were removed from the alignment, and the ML gene tree was recalculated and used as input for follow-up analyses.

To reconstruct the evolutionary history of CYP405A and CYP332A duplications, the chromosomal position of each annotated gene copy was extracted from the corresponding genome assemblies. Chromosome assignments were compared across species and interpreted together with the gene phylogenies to infer the order of duplication and loss events.

To evaluate convergent evolution in the two orthogroups *CYP405A* and *CYP332A* involved in CG biosynthesis in *Zygaena* moths and Heliconiinae butterflies, we used CSUBST (Fukushima and Pollock 2023) to derive observed nonsynonymous convergence (*O^N^_C_*), observed synonymous convergence (*O^S^_C_*), and the relative *ω_C_* metric (*O^N^_C_/E^N^_C_*)/(*O^S^_C_/E^S^_C_*), where *E^N^_C_* and *E^S^_C_* are the expected nonsynonymous and synonymous substitutions. We ran the analysis accepting all convergent events that occurred in all versus all lineage comparisons without specifying any lineages as foreground and background. For this analysis, convergence was coded as a binary variable using the CSUBST metric *ω_C any2spe_*, with branch pairs classified as convergent when *ω_C any2spe_* ≥ 1. We then evaluated whether branch pairs between *Zygaena* and Heliconiinae (assigned to the *Zygaena–*Heliconiinae category) showed an excess of convergence relative to all other branch-pair comparisons (Others-Others). Enrichment was initially assessed using Fisher's exact test on contingency tables contrasting *Zygaena–*Heliconiinae against all other comparisons. Because branch-pair statistics also depend on the evolutionary distance associated with the two branches being compared, we fitted a binomial generalized linear model with Convergence as the response, Group (*Zygaena–*Heliconiinae versus all other comparisons) as the predictor of interest, and log (dist_bl) as a covariate. Here, dist_bl is the total branch length reported by CSUBST for the branch-pair comparison. This model was used to test whether the likelihood of convergence was elevated in *Zygaena–*Heliconiinae comparisons after accounting for evolutionary opportunity. To analyze counts of convergent substitutions directly, we modeled *O^N^_C_*  _any2spe_ and *O^S^_C_*  _any2spe_ using generalized linear models with a log link and an offset of log(dist_bl), so that model coefficients could be interpreted as convergence rates per unit of evolutionary distance. We first fitted Poisson models to assess overdispersion. Because all datasets showed substantial overdispersion, we used negative binomial generalized linear models using *glm.nb* in the MASS package (Venables and Ripley 2002) for the final analyses. For each model, the effect of Group = *Zygaena–*Heliconiinae was summarized as an RR with Wald 95% CIs. All analyses were conducted in R.

Several tests for selective forces were carried out for *CYP405A* and *CYP332A* in the tribe Heliconiini: Initially, all sequences from each locus were realigned separately, and model tests and the overall *ω* (hyphy acd Universal $FASTA MG94CUSTOMCF3X4 Global) were calculated as implemented in HyPhy v2.5.32(MP) for Linux on x86_64 (Kosakovsky Pond et al. 2020) batch language. In addition, the Single-Likelihood Ancestor Counting method (Kosakovsky Pond and Frost 2005) was used to evaluate the total number of sites under purifying and positive selection, with a *P* value of 0.05 as the threshold. To detect signatures of pseudogenization, a scan for relaxed selection was initially performed for all branches of the gene trees, using RELAX.bf (Wertheim et al. 2015). Signatures of diversifying positive selection were detected by scanning all internal branches of the whole CYP405 and CYP332 phylogeny using the Adaptive Branch-Site Random Effects Likelihood method (Kosakovsky Pond et al. 2011; Smith et al. 2015) correcting for multiple tests (Bonferroni) and using a final adjusted *P* value threshold of 0.05.

Previous studies on the CG profile of heliconiines (Pinheiro de Castro et al. 2019; Sculfort et al. 2020) found that some *Heliconius* species in the Sara-Sapho clade have specialized in sequestration and might even have lost their biosynthetic ability (eg *H. sapho*, *H. antiochus*, and *H. hewitsoni*). To detect possible shifts in the selection acting upon *CYP405As* and *CYP332As* in this “sequestration-specialist” group in comparison to “plastic species,” two tests were performed: First, we applied BUSTED-PHenotype (BUSTED-PH.bf at https://github.com/veg/hyphy-analyses), a method that detects evidence of episodic diversifying selection associated with a feature, phenotype, or trait, within and between groups. This is built on BUSTED (Murrell et al. 2015) and it is implemented under HyPhy batch language. Nevertheless, because BUSTED-PHenotype does not describe the direction of the selection, we performed the RELAX (Wertheim et al. 2015) test to investigate whether the phenotype or trait branches experienced relaxation or intensification, and thus the direction of the selection.

## Supplementary Material

msag197_Supplementary_Data

## Data Availability

All data generated and analyzed in this study are publicly available either as supplementary materials in this publication or in the Apollo database in https://doi.org/10.17863/CAM.132654 or in ENA (project accession code: PRJEB72784).
